# Massive Congenital Bidirectional Coronary Arteriovenous Malformation Presenting with Signs and Symptoms of Congestive Heart Failure in an Adult: A Case Report and Review of the Literature

**DOI:** 10.1155/2011/186921

**Published:** 2011-09-29

**Authors:** H. M. M. Al Hashimi, A. J. Wardeh, S. Bulut, F. W. A. Verheugt

**Affiliations:** ^1^Canisius Wilhelmina Hospital, P.O. Box 9015, 6500 GS Nijmegen, The Netherlands; ^2^MCH Westeinde Hospital, P.O. Box 432, 2501 CK Den Haag, The Netherlands; ^3^Gelre Hospital Zutphen, P.O. Box 9020, 7200 GZ Zutpen, The Netherlands; ^4^Onze Lieve Vrouwe Gasthuis Hospital, P.O. Box 95500, 1090 HM Amsterdam, The Netherlands

## Abstract

Congenital anomalies of the coronary arteries are relatively rare. Mostly asymptomatic, however, some can cause problems, as heart failure, myocardial ischemia, and ventricular arrhythmia, and are associated with risk of complications, such as endocarditis and coronary rupture or sudden death. A case of a 69-year-old man with complaints of tiredness, dyspnea, and palpitation due to coronary artery fistula is presented with a review of the literature.

## 1. Case Report

A 69-year-old man consulted a cardiologist because of progressively increased tiredness, dyspnea, and palpitation during the last year. He had no complaints of angina. Physical examination revealed a slightly elevated jugular pressure, and a systolic murmur at the apex radiating to the axilla. His electrocardiogram showed atrial fibrillation, normal axis, and a nonspecific interventricular conduction defect resembling incomplete right bundle branch block. Chest X-ray was suggestive for cardiomegaly with atrial enlargement. 

Transthoracic echocardiogram confirmed left and right atrial enlargement, with preserved left ventricular function, moderate mitral valve incompetence, and severe tricuspid valve incompetence. A blunted inspiratory collapse of the inferior caval vein indicated increased right atrial pressure.

**Figure 1:**
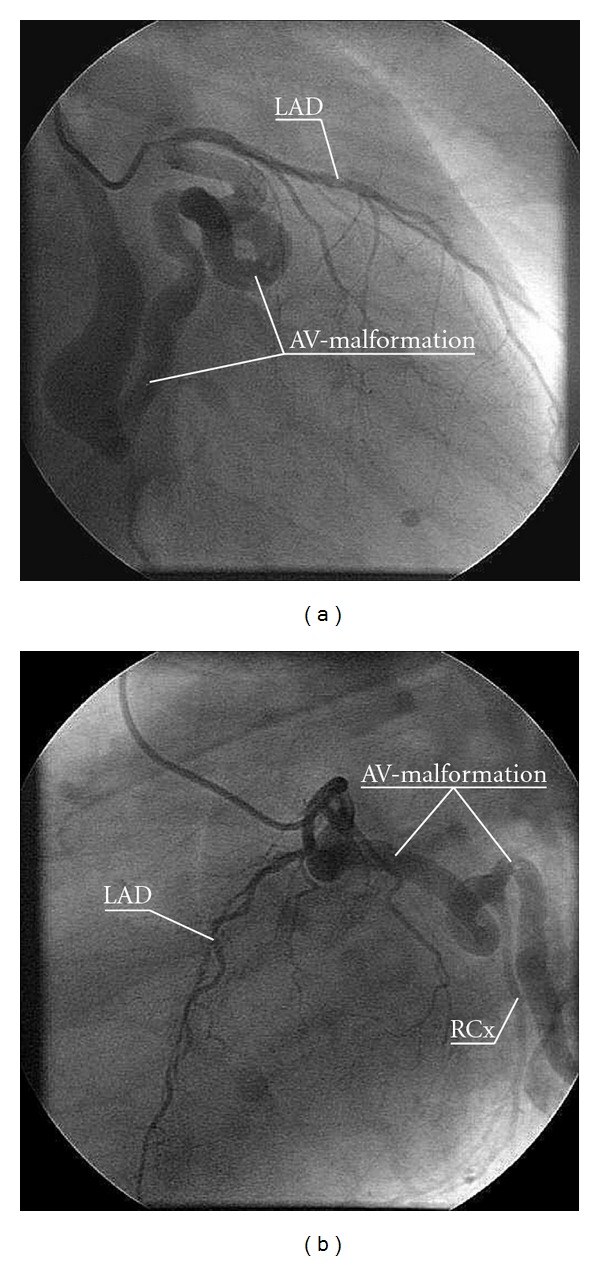
(a) Coronary arteriography showing a large and tortuous arteriovenous malformation originating from the left coronary sinus with a massive venous ectasia draining into the right atrium and vena cava. AV = arteriovenous. LAD = left anterior descending artery. (b) There is an aberrant circumflex artery originating from the arteriovenous malformation. AV = arteriovenous. RCx = ramus circumflex artery. LAD = left anterior descending artery.

Coronary angiography revealed a large, tortuous fistula originating from the left aortic sinus and draining into the right atrium. An aberrant circumflex artery originated from this fistula (Figures 1(a) and 1(b)). 

**Figure 2:**
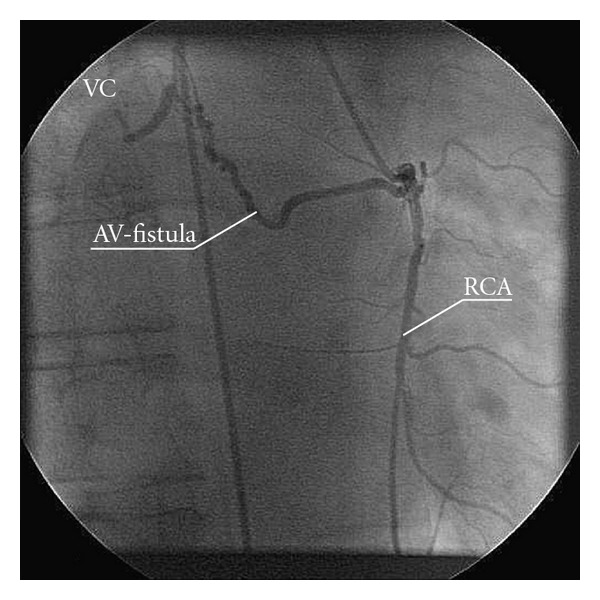
Arteriovenous fistula originating from the right coronary artery and draining into the vena cava. VC = vena cava. RCA = right coronary artery. AV = arteriovenous.

A second, but smaller fistula originated from the right coronary and drained into the superior cava vein was also detected during angiography (Figure 2). 

**Figure 3:**
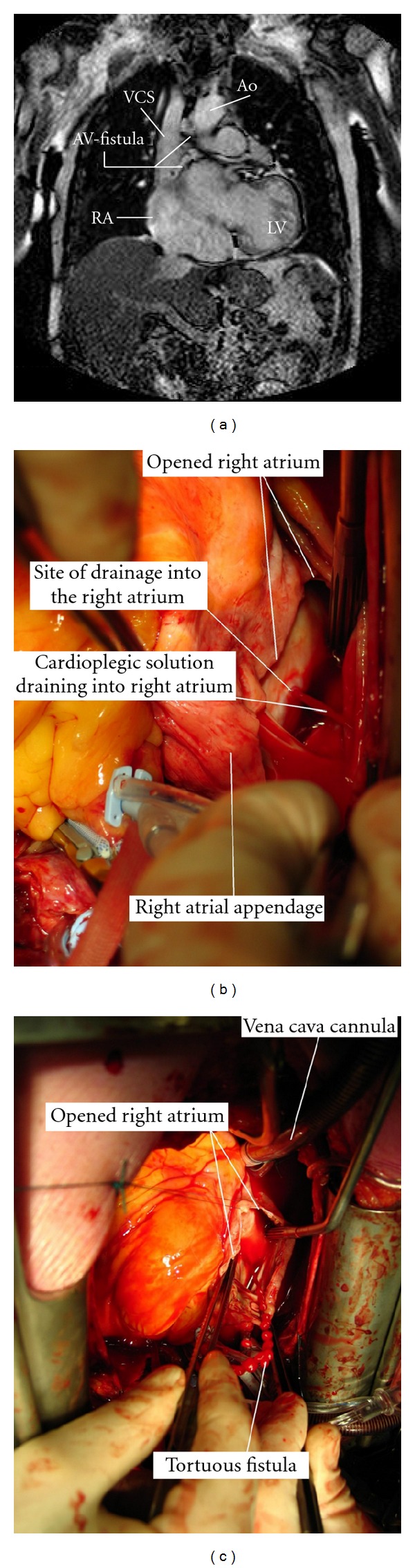
(a) A MRI image showing the arteri-venous malformation originating from the left coronary sinus and draining into the right atrium. Ao = aorta. VCS = vena cava superior. AV = arterio-venous. RA = right atrium. LV = left ventricle. (b) Intra operative image with the site of drainage into the right atrium. (c) Intra-operative image showing the very tortuous arterio-venous fistula.

Magnetic resonance imaging scan confirmed this large malformation (Figure 3(a)). 

These findings were also confirmed during open heart surgery. See Figures 3(b) and 3(c).

Patient was operated through median sternotomy, using cardiopulmonary bypass. The arteriovenous malformations were ligated, and a mitral and tricuspid valve repair was performed in combination with an MAZE procedure. 

The patient was discharged home and was seen at the outpatient clinic. He was doing well without any complaints.

## 2. Discussion

A coronary artery fistula is an abnormal communication, shunting blood, between a coronary artery and a cardiac chamber, major vessel (vena cava and pulmonary artery) or another vascular structure as coronary sinus and mediastinal vessels. Most fistulae are due to congenital cause, but some are acquired, postcardiac traumata, myocardial infarction, and even cardiac surgery or catheter intervention [1, 2]. Krause published a first pathologic report concerning this anomaly in 1865 [3]. 

The real incidence is unknown, because most fistulae are small and asymptomatic. In patients referred for coronary angiography, the incidence is ranging from 0.1 to 0.2%. Fistulae can originate from left and right coronary artery, however, in about 60% from the right coronary artery. Nearly 90% of the fistulae empty into a right heart low-pressure chamber. Bilateral fistulae are rare, totalling 4%-5% of the reported arteriovenous fistulae; 50%–60% of them terminate in the pulmonary artery, in contrast to the unilateral fistulae (20%) [1, 2, 4, 5]. The etiology of coronary fistulae is unclear. It is suggested that they are the result of an anomalous development of intratrabecular spaces between the lumens of the tubular heart during intrauterine life. Normally, these spaces shrink immediately after birth and become capillaries or thebesian vessels [6]. This theory can explain fistulas between coronary arteries and cardiac chambers. Fistulae resulting from abnormal development of the coronary vessels is another theory concerning coronary fistulae. The first embryonic evidence of coronary vessel development is the appearance of blood islands just under the epicardium of the developing heart during the beginning of the fifth week. During the late fifth and sixth week, the capillary plexuses developing from these foci form connections birth with coronary veins sprouting from the coronary sinus and with coronary arteries growing from the aortic sinuses [7]. This theory can explain fistula as described in our case, like a single vessel with a single site of origin and termination.

Most fistulae are small and asymptomatic. They are only discovered incidentally during coronary angiography or during screening for an asymptomatic cardiac murmur. However, fistulae can be symptomatic, particularly larger fistulae. Several complaints and complications are described such as congestive heart failure, dyspnea, arrhythmias, myocardial ischemia/infarction, pulmonary hypertension, infectious endocarditis, aneurysm formation, coronary rupture, and death. The pathophysiology thought to be myocardial stealing or reduction in myocardial blood flow distal to the site of the fistula. The mechanism is related to the diastolic pressure gradient and runoff from the coronary vasculature to the low-pressure receiving cavity. If the fistula is large, the intracoronary diastolic perfusion pressure diminishes progressively. Because fistulae enlarge over the time, this can explain why only 10% of patients younger than 20 years have complaints. Patients older than 20 years have complaints in 35% of cases [1, 2, 8–10]. 

As already mentioned, most fistulae are accidentally detected during routine coronary angiography. Clinical diagnosis is usually suspected from detection of a continuous cardiac murmur. Chest X-ray and electrocardiogram are normal if the shunt throught the fistula is small but may show evidence of cardiomegaly, left ventricular hypertrophy, and atrial enlargement depending on the extend of the shunt. Coronary artery angiography and aortography is, of course, the gold standard for the diagnosis. Echocardiography may reveal atrial or ventricular enlargement as a consequence of the shunt, decreased or dysfunction of the left ventricle due to myocardial ischemia, and even be helpful in detecting entrance and termination of the shunt. Also, computed tomography imaging of the chest can visualize fistula and enlargement of heart chambers [1, 2, 5, 8, 11]. 

Despite the fact that spontaneous closure of a fistula is uncommon, it is generally accepted that small asymptomatic fistulae do not need therapeutic intervention. Surgical or catheter intervention is, however, recommended for symptomatic patients and patients at risk for complications [1, 2, 8, 10, 12]. Infective endocarditis, thromboembolic events and even rupture are described [1, 2, 10, 12]. 

Percutaneous intervention with transcatheter embolization is the treatment of choice with low morbidity rates. But it is a complicated intervention requiring an interventional specialist with expertise in coronary angiography and embolization techniques [13–15]. Multiple fistulae, multiple drainage sites, presence of large branch vessels, or difficulties in accessing the coronary artery supplying the fistula are exclusion criteria for transcatheter coil embolization.

A general technique for surgical correction does not exist. Ligation with or without cardiopulmonary bypass is the simplest surgical intervention. However, surgical treatment should be adapted to the anatomy of the fistula. Normal coronary circulation might have to be reconstructed. Surgery-related complications to the ligation of fistulae are low. In cases of giant coronary aneurysm and additional cardiac surgical procedures, the risk is, however, increased [2, 8, 12, 16].
